# Factors influencing cognitive function in patients with Huntington's disease from China: A cross‐sectional clinical study

**DOI:** 10.1002/brb3.3258

**Published:** 2023-10-17

**Authors:** Yang‐Fan Cheng, Kun‐Cheng Liu, Tian‐Mi Yang, Yi Xiao, Qi‐Rui Jiang, Jing‐Xuan Huang, Sirui Zhang, Qian‐Qian Wei, Ru‐Wei Ou, Chun‐Yu Li, Xiao‐Jing Gu, Jean‐Marc Burgunder, Hui‐Fang Shang

**Affiliations:** ^1^ Department of Neurology Laboratory of Neurodegenerative Disorders Rare Disease Center West China Hospital Sichuan University Chengdu China; ^2^ Swiss Huntington's Disease Centre, Siloah, Department of Neurology University of Bern Bern Switzerland

**Keywords:** Chinese population, cognitive function, Huntington's disease, influencing factors

## Abstract

**Background and aim:**

Huntington's disease (HD) is an autosomal dominant inherited neurodegenerative disorder caused by CAG repeats expansion. Cognitive decline contributes to the loss of daily activity in manifest HD. We aimed to examine the cognition status in a Chinese HD cohort and explore factors influencing the diverse cognitive domains.

**Methods:**

A total of 205 participants were recruited in the study with the assessment by neuropsychological batteries, including the mini–mental state examination (MMSE), Stroop test, symbol digit modalities test (SDMT), trail making test (TMT), verbal fluency test (VFT), and Hopkins verbal learning test–revised, as well as motor and psychiatric assessment. Pearson correlation and multiple linear regression models were applied to investigate the correlation.

**Results:**

Only 41.46% of patients had normal global function first come to our center. There was a significantly difference in MMSE, Stroop test, SDMT, TMT, and VFT across each stage of HD patients (*p* < .05). Apathy of PBA‐s was correlated to MMSE, animal VFT and Stroop‐interference tests performance. Severity of motor symptoms, functional capacity, age, and age of motor symptom onset were correlated to all neuropsychological scores, whereas education attainment and diagnostic delay were correlated to most neuropsychological scores except TMT. Severity of motor symptoms, functional capacity, and education attainment showed independent predicting effect (*p* < .05) in diverse cognitive domains.

**Conclusion:**

Cognitive impairment was very common in Chinese HD patients at the first visit and worse in the patients in advanced phase. The severity of motor symptoms and functional capacity were correlated to the diverse cognitive domains.

## Graphical abstract

We found that only 41.46% of patients had a normal global function on their first visit to our center with the median disease duration of 4.5 years. There was a significantly increased decline in diverse cognitive domains tested by mini–mental state examination (MMSE), Stroop, symbol digit modalities test (SDMT), trail making test (TMT), and verbal fluency test (VFT) tests across each stage of HD patients (*p* < .05). Severity of motor symptoms, functional capacity, age, and age of motor symptom onset were correlated to all neuropsychological scores.

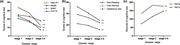

## 1 INTRODUCTION

Huntington's disease (HD) is an autosomal‐dominant, progressive neurodegenerative disorder with the characteristic of a triad of progressive motor abnormalities, psychiatric symptoms, and cognitive impairment (McColgan & Tabrizi, 2018). HD is caused by an expanded CAG repeat tract of at least 36 trinucleotides in the huntingtin *(HTT)* gene on chromosome 4, which encodes an expanded polyglutamine stretch in HTT protein (Tabriz et al., 2022). Typically, the onset of symptoms usually appears in middle age, after affected individuals have already had children. However, the disorder could manifest at any point in life, ranging from infancy to senescence (Bates et al., 2015; Podvin et al., 2019). No disease‐modifying treatment is available hitherto, and premature death typically occurs 10–20 years after the initial onset of symptoms, which is typically characterized by chorea (Bates et al., 2015; Dorsey et al., 2013).

Cognitive impairment is one of the triad of symptoms in manifest HD, representing impairments in attention, verbal fluency, psychomotor speed, executive functioning, memory, and visuospatial functioning (Duff et al., 2010; Ross et al., 2014), which may appear even in gene‐positive individuals before the onset of motor symptoms (Papoutsi et al., 2014; Peavy et al., 2010). The cognitive impairment is progressive and a contributing factor for daily disability, which is generally considered to be more debilitating to the patients and their families (Beerens et al., 2013; Horta‐Barba et al., 2022; Papoutsi et al., 2014). During the early stage, clinicians may overlook subtle cognitive impairment in HD patients (Julayanont et al., 2020). Additionally, the assessment of cognition in these patients could be challenging due to the diverse range of associated symptoms, including difficulties in interpreting cognitive tasks in individuals with motor dysfunction. Therefore, it is crucial to utilize multidimensional and comprehensive neuropsychiatric batteries to accurately understand the cognitive status of HD patients. However, few studies on cognitive function in HD patients from China have been reported.

Therefore, the aim of the study was to assess the cognitive status in a Chinese HD cohort through the use of various neuropsychiatric batteries. Additionally, we aimed to investigate the influence of sociodemographic and clinical factors, including motor and neuropsychiatric symptoms, on the assessment of diverse cognitive domains to identify more independent factors of cognitive capacity.

## 2 METHODS

### 2.1 Participants

We recruited 205 symptomatic, gene‐confirmed (CAG >39) HD patients (84 males and 121 females). from the Department of Neurology of West China Hospital. The participants of this study was based on the same cohort as the previous study (Cheng et al., 2022). The current study expanded upon the comprehension of the cohort's attributes and explored cognitive aspects that were not encompassed in the earlier investigation. All the clinical tests and neurological examinations were administered by experienced and trained neurologists. Participants were excluded from the study if they had any neurological disorder other than HD, a history of head trauma, epilepsy, drug abuse, non‐corrected visual problems, active major psychotic or delusional syndrome, or severe language difficulties. This study adhered to the principles of the Declaration of Helsinki and was approved by the Ethics Committee of West China Hospital (approval number 2015‐236). All participants signed the informed consent.

### 2.2 Assessment of neuropsychological functioning

In the current study, to get a comprehensive as well as a precise picture of cognitive status in patients with HD, we conducted a series of neuropsychological assessments including mini–mental state examination (MMSE) (Folstein et al., 1975), verbal fluency test (VFT) (Boll et al., 1974; Swanson, 2005) including semantic and letter, symbol digit modalities test (SDMT) (Smith, 1973), Stroop test (Kremer & Group, 1996), trail making test (TMT) A&B (Bowie & Harvey, 2006), and Hopkins verbal learning test–revised (HVLT‐R) (Brandt & Benedict, 2001) to assess neuropsychological function, which covered five cognitive domains including executive function: letter VFT (Swanson, 2005), TMT‐Part B, Stroop‐interference test; psychomotor speed and attention: SDMT (Smith, 1973); processing speed: Stroop‐word reading, Stroop‐color naming, TMT‐Part A; semantic categorical language function: animal fluency test; and episodic memory: HVLT‐R. The selection of cognitive tasks was based on their inclusion in standard assessment batteries of the Unified Huntington's Disease Rating Scale (UHDRS) and extensively employed to evaluate broader cognitive skills and a range of executive functions (Eddy & Rickards, 2015; Julayanont et al., 2020).

The MMSE has been tested as a cognitive screening test for use in HD (Landwehrmeyer et al., 2017; Ringkøbing et al., 2020), which comprises 11 questions spanning 5 aspects of cognitive function: executive function, language, memory function, visuospatial ability, and orientation (range 0–30 points) with good inter‐rater, test, and retest reliability in differentiating cognitive status in disorders featuring cognitive impairment (Godefroy et al., 2011). The VFT test includes animal and letter VFT (Swanson, 2005). In animal VFT, participants have to produce as many words as possible from a category of animal in 60 s. Moreover, in letter VFT, participants have to produce as many words as possible beginning with Chinese characters “Tian,” “Yue,” and “Hao” (Boll et al., 1974; Liu et al., 2022). Paper‐version SDMT (Smith, 1973) is a simple substitution task, which gives the participants 90 s to pair specific numbers with given geometric figures with written responses. The Stroop test (Kremer & Group, 1996) includes three timed conditions that measure the speed of processing and the ability to inhibit competing responses. Color naming requires naming the colors of blocks presented horizontally. Word reading requires reading color words printed in black ink, and the interference condition, naming the ink color of color words while inhibiting word reading. The number of correct responses (including corrected responses) in 45 s determines the score in each condition. TMT requires a participant to connect a sequence of 25 consecutive targets on a sheet of paper, similar to a child's connect‐the‐dots puzzle. There are two parts to the test: in part A, the targets are all numbers from 1 to 25 and the test taker needs to connect them in sequential order; in part B, the dots go from 1 to 13 and include Chinese characters corresponding to A–L. As in the first part, the patient must connect the dots in order while alternating characters and numbers, as in 1‐A‐2‐B‐3‐C…, in the shortest time possible without lifting the pen from the paper (Bowie & Harvey, 2006). Scoring is based on the time taken to complete the test. HVLT‐R was used to assess episodic memory (Brandt & Benedict, 2001). In this test, participants are given 3 trials to learn a list of 12 related words. After a 20‐min delay, free recall for the 12 words is assessed. The raw score uses the sum of the total recall and delayed free recall (number correct).

### 2.3 Assessment of motor, functional capacity, and neuropsychiatric symptoms

Sociodemographic information, including sex, age, ethnicity, education attainment (years), and family history, and clinical information, including age of motor symptom onset (AAO), CAG repeat length, disease duration (from the time of initial symptom to the point of this clinical interview), and diagnostic delay (duration from onset to diagnosis), were collected by professional neurologists in a face‐to‐face interview. Every interview included the administration of a battery of cognitive, behavioral, motor, and functional assessments.

The UHDRS–total motor score (TMS) and the total functional capacity (TFC) score (Shoulson et al., 1989) were used to assess the motor symptom and to quantify a patient's ability to perform basic and instrumental activities of daily living as described previously (Cheng et al., 2022). Participants were divided into two groups, which were sever motor symptom (TMS score more than mean score) and mild motor symptom (TMS score less than mean score). The participants were grouped into five stages in the current study based on their TFC score: stage 1 = TFC of 11–13; stage 2 = TFC of 7–10; stage 3 = TFC of 3–6; stage 4 = TFC of 1–2; and stage 5 = TFC of 0 (Shoulson, 1981). The disease burden score was calculated using the formula CAP = ([CAG − 35.5] × age), which had the quotient of the degree of atrophy in the striatum (the brain region most severely affected in HD (Penney et al., 1997)) to explore whether associations between cognition and pathologic stage of disease.

Behavioral symptoms were assessed using the short form of the problem behavior assessment for HD (PBA‐s) (Callaghan et al., 2015). The PBA‐s measured severity and frequency of 11 behavioral problems and psychiatric symptoms, including depression, suicidal ideation, anxiety, anger/aggression, irritability, apathy, obsessive compulsive behavior, perseverative thinking, delusions, hallucinations, and disoriented behavior (Callaghan et al., 2015; Martinez‐Horta et al., 2016). A sub‐score of each item was computed by multiplying the severity score and frequency score of that item. Total scores for each subscale were computed by summing the total scores of the separate items of each subscale (Cheng et al., 2022). Participants were divided into three groups, which were no, mild, and moderate‐to‐severe symptoms, depending on their total subscale score (Cheng et al., 2022). Additionally, we also used the Hamilton Depression Scale (HAMD; 24 items) (Hamilton, 1967) and Beck Depression Inventory (BDI) (Beck et al., 1961), which are widely used and recognized scales for assessing depression based on reliability and familiarity among researchers and clinicians to assist the assessments of neuropsychiatric symptoms. Considering depression is common symptoms during the course of HD, we used HAMD and BDI scales to assess the severity of depression more specific to explore whether the correlation between cognitive function and depression. Participants were divided into three groups, which were no symptoms (HAMD <8 scores or BDI <4 scores), mild symptoms (HAMD: 8–20 scores or BDI: 5–7 scores), and moderate‐to‐severe symptoms (HAMD >20 scores or BDI >8 scores).

The interview procedure following confirmation of the genetic test involved gathering sociodemographic and clinical information. Subsequently, we conducted assessments in the following order: neuropsychological tests, UHDRS, and PBA‐s. To ensure accurate results during the neuropsychological tests, it was essential to secure patients’ complete focus. The interviews took place in individual, quiet consulting rooms to create an optimal testing environment.

### 2.4 Statistical analysis

The mean ± standard deviation and the *t*‐test were used to analyze data that conformed to the normal distribution. Multivariate ANOVA analyses were used to compare the cognitive assessment of patients in different disease stages adjusted by age and education attainment. To compare the strength of the relationship between continuous variables, Pearson correlation analysis was used.

To avoid confounders, baseline variables that were considered clinically relevant or that showed a univariate relationship with outcome were entered into multiple linear regression model, which was used to determine the influencing factors of cognitive function in patients with HD, using the total score of each domain as a dependent variable and the patient's sex, CAG repeats, AAO, family history, education attainment, TMS, TFC, and subdomain scores of PBA‐s as independent variables. These univariate tests included simple Pearson's rho correlations and Student's *t*‐tests (Q–Q plots were inspected to ensure normality assumption) between cognition score and the other putative predictors. Sex and age were considered clinically relevant confounders and forced enter all the models. Standardized beta coefficients and *p* values were reported, along with the adjusted *R* ^2^ values denoting the overall percentage of the variance of cognitive functions explained by predictors. Adjusted *R* ^2^ values between .01 and .08 were reported to be minimal effect sizes, values between .09 and .24 were considered moderate, and effect sizes greater than .25 were large (Fritz et al., 2018). All assumptions of linear models were checked (e.g., linearity, homoscedasticity, auto‐correlation, and multicollinearity). Statistical analysis was conducted using Prism GraphPad and SPSS 22.0. Moreover, *p* < .05 or *p* < .05/*n* (Bonferroni correction) was considered statistically significant.

## 3 RESULTS

### 3.1 The demographic characteristics of patients

This cross‐sectional study enrolled 205 symptomatic, gene‐confirmed (CAG >39) HD patients (84 males and 121 females). The mean age of participants was 52.92 ± 11.54 years old, and the estimated median disease duration was 4.5 years (interquartile range [IQR]: 2.34–6.92). The median CAG repeats numbers were 44 (IQR: 42–48), with the mean motor AAO of 41.81 ± 9.99 (range 17–69) years old. More detailed information could be found in the previously published study (Cheng et al., 2022) and Table S1.

### 3.2 Results of the cognitive assessments

**FIGURE 1:**
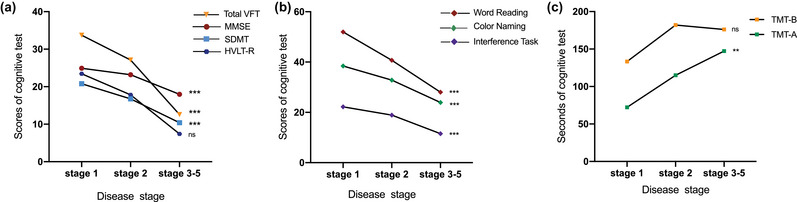
The neuropsychological tests grouped by disease stages. (a) The test of total verbal fluency test (VFT) score, mini–mental state examination (MMSE), symbol digit modalities test (SDMT) were getting worse among patients in different disease stages after age and education attainment adjusted (*p* < .001), whereas total recall in Hopkins verbal learning test–revised (HVLT‐R) did not have statistical significance. (b) Three parts in Stroop test, including word reading, color naming, and interference task became worse with the disease progression (*p* < .001); (c) TMT‐A showed the statistically significant decline among different disease stages. *p < .05; **p < .01; ***p < .001; ns: *p* > .05.

The mean score of MMSE in patients of stage 1 and 2 was 23.99 ± 4.42 points, whereas the score in advanced stage (3–5) was 17.98 ± 5.15 points. Only 41.46% (reported in 68/164) HD patients had normal MMSE score (25–30 points). There was a significant difference in scores of MMSE (*p* < .001), VFT (*p* < .001), SDMT (*p* < .001), Stroop test (*p* < .001) including color naming (*p* < .001), word reading (*p* < .001), interference task (*p* < .001), and TMT test (*p* = .02) across HD stages after age and education attainment adjustment (Table 1 and Figure 1), whereas no statistical significance was found in HVLT‐R and TMT‐B. No statistical significance was found in CAP scores among different disease stages. The detailed scores of motors, neuropsychiatric, and cognition assessments are shown in Table S2.

**TABLE 1:** Neuropsychological assessment grouped by disease stage.

	Stage 1 (TFC: 11–13)	Stage 2 (TFC: 7–10)	Stage 3–5 (TFC: 0–6)	*p* Value
Age (years old)	50.95 ± 11.46	52.60 ± 11.02	56.46 ± 11.70	.028^a^
Education attainment (years)	9.65 ± 4.18	10.21 ± 4.31	7.80 ± 4.08	.007^a^
CAG products (CAPs)	100.3 ± 18.37	103.0 ± 15.68	105.6 ± 21.27	.37
MMSE	25.41 ± 3.50	23.20 ± 4.40	17.58 ± 5.15	<.001^b^
VFT total	33.71 ± 10.36	27.11 ± 8.96	12.57 ± 5.50	<.001^b^
Letter fluency test	20.79 ± 9.21	16.76 ± 6.49	6.21 ± 4.72	.001^b^
Animal fluency test	12.92 ± 6.81	10.35 ± 3.69	6.40 ± 1.72	.001^b^
SDMT	23.45 ± 11.58	17.12.57 ± 8.58	7.42 ± 6.32	<.001^b^
Stroop test total	112.6 ± 39.98	91.87 ± 33.49	62.32 ± 25.77	<.001^b^
Color naming	38.43 ± 15.29	32.81 ± 13.91	23.93 ± 9.35	<.001^b^
Word reading	51.97 ± 17.34	40.70 ± 15.54	28.00 ± 13.16	<.001^b^
Interference task	22.18 ± 11.82	18.91 ± 9.30	11.55 ± 9.78	<.001^b^
Trail making task (s)	205.6 ± 102.6	299.2 ± 94.59	320.7 ± 107.9	.02^b^
TMT‐A	72.23 ± 38	115.0 ± 49.26	147.3 ± 55.21	.003^b^
TMT‐B	133.4 ± 70.81	182.0 ± 63.09	176.1 ± 64.58	.11
HVLT‐R	20.79 ± 9.21	16.76 ± 6.49	10.40 ± 5.94	.95
Total recall	17.00 ± 6.10	14.18 ± 4.46	8.80 ± 3.77	.94
Delayed free recall	6.55 ± 3.39	4.40 ± 3.24	2.67 ± 2.52	.98

Abbreviations: HVLT‐R, Hopkins verbal learning test–revised; MMSE, mini–mental state examination; SDMT, symbol digit modality test; TMT, trail making task; TFC, total functional capacity scale; VFT, verbal fluency test.

^a, b^ Statistically significant among different disease stages.

^b^ *p* Value has adjusted by age and education attainment.

### 3.3 Correlation analyses and multiple regression analysis of cognition

First, we conducted Pearson correlation analyses to find the variables of sociodemographic and clinical features correlated with the performance of neuropsychological tests. We found that the score of MMSE was significantly correlated with the educational attainment (*r* = .536), TFC (*r* = .528) and the dysexecutive part of PBA‐s (*r* = −.441) (*p* < .001) (for the sake of article length, we listed the top three factors correlated with the score of cognitive test); the animal VFT test was significantly correlated with TMS (*r* = −.459), education attainment (*r* = .398), and TFC (*r* = .387) (*p* < .001); the letter VFT test was significantly correlated with education attainment (*r* = .479), TMS (*r* = −.447), and TFC (*r* = .348) (*p* < .001); the TMT‐A test was significantly correlated with TMS (*r* = .572), TFC (*r* = −.460), and age (*r* = .448) (*p* < .001); the TMT‐B test was significantly correlated with TMS (*r* = .355), TFC (*r* = −.284), and HAMD (*r* = .260) (*p* < .001); the Stroop naming test was significantly correlated with TMS (*r* = −.532), TFC (*r* = .401), and education attainment (*r* = .398) (*p* < .001); the Stroop reading test was significantly correlated with TMC (*r* = −.562), TFC (*r* = .498), and age (*r* = −.432) (*p* < .001); the Stroop interfere test was significantly correlated with TMS (*r* = −.467), diagnostic delay (*r* = −.389), and TFC (*r* = .296) (*p* < .001); the SDMT was significantly correlated with TFC (*r* = .619), TMS (*r* = −.609), and age (*r* = −.445) (*p* < .001); HVLT‐R were significantly correlated with CAG repeats (*r* = .522), age (*r* = −.486), and education attainment (*r* = .452) (*p* < .001). More detailed information is shown in Table 2.

**TABLE 2:** Pearson's *r* (and related level of significance) between variables on basic and clinical features and cognitive test performance.

		Sex	Age	CAG repeats	CAPs	Diagnostic delay	AAO	Education attainment	Family history	TMS	TFC	Affect	Irritability	Apathy	Dysexecutive	Psychosis	HAMD	BDI
**MMSE**	Pearson r	0.117	−0.324	0.235	0.076	−0.401	−0.180	0.536	−0.105	−0.428	0.528	−0.110	−0.150	−0.214	−0.441	0.071	−0.215	−0.061
	*p* Value	.134	**<.001** ^a^	**.002** ^a^	.336	**<.001** ^a^	**.021** ^a^	**<.001** ^a^	.179	**<.001** ^a^	**<.001** ^a^	.178	.064	**.008** ^a^	**<.001** ^a^	.382	**.029** ^a^	.581
**Animal VFT**	Pearson r	−0.065	−0.352	0.327	0.168	−0.347	−0.254	0.398	0.135	−0.459	0.387	0.196	−0.010	−0.271	−0.170	−0.013	0.011	0.044
	*p* Value	.577	**.002** ^a^	**.004** ^a^	.144	**.002** ^a^	**.026** ^a^	**<.001** ^a^	.240	**<.001** ^a^	**<.001** ^a^	.088	.929	**.017** ^a^	.139	.914	.926	.741
**Letter VFT**	Pearson r	0.038	−0.308	0.166	0.001	−0.229	−0.118	0.479	−0.079	−0.447	0.348	−0.039	−0.144	−0.152	−0.149	−0.038	−0.123	−0.192
	*p* Value	.630	**<.001** ^a^	**.032** ^a^	.994	**.003** ^a^	.123	**<.001** ^a^	.313	**<.001** ^a^	**<.001** ^a^	.641	.081	.065	.071	.650	.270	.108
**Trail making test A**	Pearson r	−0.063	0.448	−0.360	−0.124	0.322	0.409	−0.379	0.030	0.572	−0.460	0.008	−0.043	0.157	0.243	−0.001	0.152	0.080
	*p* Value	.603	**<.001** ^a^	**.002** ^a^	.304	**.006** ^a^	**<.001** ^a^	**.002** ^a^	.803	**<.001** ^a^	**<.001** ^a^	.946	.723	.191	**.041** ^a^	.989	.212	.549
**Trail making test B**	Pearson r	−0.199	0.167	−0.116	0.051	0.112	0.208	−0.159	−0.022	0.355	−0.284	0.126	−0.218	0.097	0.001	0.002	0.260	0.140
	*p* Value	.103	.174	.348	.678	.365	.089	.209	.858	**.003** ^a^	**.019** ^a^	.307	.074	.431	.998	.989	**.035** ^a^	.309
**Stroop naming**	Pearson r	−0.137	−0.334	0.265	0.115	−0.333	−0.199	0.398	−0.175	−0.532	0.401	−0.0653	−0.150	−0.0866	−0.247	−0.0374	−0.212	−0.0952
	*p* Value	.082	**<.001** ^a^	**<.001** ^a^	.143	**<.001** ^a^	**.011** ^a^	**<.001** ^a^	**.025** ^a^	**<.001** ^a^	**<.001** ^a^	.431	.068	.296	**.003** ^a^	.651	.056	.423
**Stroop reading**	Pearson r	−0.133	−0.432	0.331	0.117	−0.391	−0.300	0.328	−0.0588	−0.562	0.498	−0.0316	−0.103	−0.200	−0.262	−0.0336	−0.207	−0.159
	*p* Value	.099	**<.001** ^a^	**<.001** ^a^	.145	**<.001** ^a^	**<.001** ^a^	**<.001** ^a^	.466	**<.001** ^a^	**<.001** ^a^	.708	.222	.017	**.002** ^a^	.690	.064	.184
**Stroop interference**	Pearson r	−0.123	−0.168	0.169	0.103	−0.389	−0.0271	0.236	−0.0787	−0.467	0.296	−0.151	−0.259	−0.191	−0.206	−0.103	−0.334	−0.256
	*p* Value	.131	**.038** ^a^	**.037** ^a^	.209	**<.001** ^a^	.741	**.004** ^a^	.335	**<.001** ^a^	**<.001** ^a^	.078	**.002** ^a^	**.025** ^a^	**.015** ^a^	.228	**.003** ^a^	**.036** ^a^
**SDMT**	Pearson r	−0.062	−0.445	0.202	0.387	0.386	−0.355	0.359	0.116	−0.609	0.619	0.119	−0.165	−0.117	−0.320	−0.081	−0.188	−0.139
	*p* Value	.312	**<.001** ^a^	.054	**<.001** ^a^	**.001** ^a^	**.002** ^a^	**.002** ^a^	.178	**<.001** ^a^	**<.001** ^a^	.173	.094	.177	**.005** ^a^	.261	.067	.134
**HVLT‐R**	Pearson r	0.026	−0.486	0.522	0.132	−0.441	−0.376	0.452	0.177	−0.310	0.402	0.068	−0.105	−0.259	−0.186	0.192	−0.109	−0.020
	*p* Value	.438	**.001** ^a^	**<.001** ^a^	.832	**.002** ^a^	**.009** ^a^	**.002** ^a^	.140	**.028** ^a^	**.006** ^a^	.341	.263	.056	.128	.120	.254	.452

*Note*: Bold *p* values (<.05) are statistically significant.

Abbreviations: BDI, Beck Depression Inventory; CAPs, CAG‐age‐product score; HAMD, Hamilton depression scale; HVLT‐R: Hopkins verbal learning test–revised; MMSE, mini–mental state examination; PBA‐s, short version of problem behavior assessment; SDMT, symbol digit modality test; TFC, total functional capacity scale; TMS, total motor score in the unified Huntington's disease rating scale; TMT, trail making task.

**FIGURE 2:**
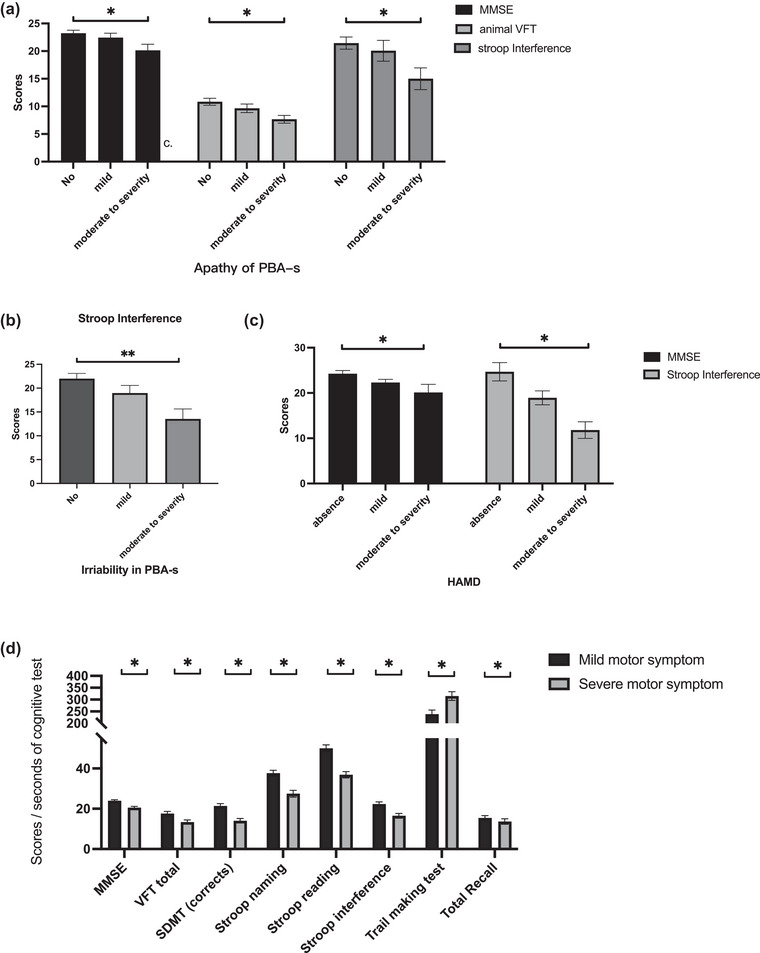
The comparison of cognitive tests grouped by severity of neuropsychiatric and motor symptoms. On apathy, the scores of mini–mental state examination (MMSE) (*p* = .019), animal verbal fluency test (VFT) (*p* = .038), and Stroop‐interference (*p* = .046) showed statistical difference among participants with no, mild, and moderate‐to‐severe symptoms. More specific, participants with no symptoms of apathy had higher performance on these tests (MMSE: *p* = .014; animal VFT: *p* = .033; Stroop‐interference: *p* = .036) than participants with moderate‐to‐severe symptoms (*p* < .05) (a). Similarity, participants with no symptoms of irritability had higher performance on Stroop‐interference (*p* = .005) than participants with moderate‐to‐severe symptoms (b). The scores of MMSE (*p* = .041) and Stroop‐interference (*p* = .011) showed statistical difference among participants with no, mild, and moderate‐to‐severe symptoms according to the Hamilton Depression Scale (HAMD) scores (c). There were significant difference between two groups on the scores of MMSE, total VFT, symbol digit modalities test (SDMT), Stroop reading/naming/interference, trail making test (TMT), and HVAL‐R (*p* < .05) (d).

Then, we conducted the analyses grouped by severity of neuropsychiatric and motor symptoms according to the Pearson correlation coefficient. Participants were divided into three groups, which were no , mild, and moderate‐to‐severe symptoms, depending on their total subscale score of PBA‐s. On apathy, we found that the scores of MMSE (*p* = .019), animal VFT (*p* = .038), and Stroop‐interference (*p* = .046) showed statistical difference among participants with no , mild, and moderate‐to‐severe symptoms. More specific, participants with no symptoms of apathy had higher performance on these tests (MMSE: *p* = .014; animal VFT: *p* = .033; Stroop‐interference: *p* = .036) than participants with moderate‐to‐severe symptoms (*p* < .05) (Figure 2a). Similarity, participants with no symptoms of irritability had higher performance on Stroop‐interference (*p* = .005) than participants with moderate‐to‐severe symptoms (Figure 2b). Because there were few participants with mild and moderate‐to‐severe dysexecutive, we did not compare the severity of different groups with dysexecutive and cognitive tests. Additionally, we also grouped participants based on HAMD and BDI scores. We found that the scores of MMSE (*p* = .041) and Stroop‐interference (*p* = .011) showed statistical difference among participants with no, mild, and moderate‐to‐severe symptoms according to the HAMD scores, whereas no significant difference was found based on BDI scores (*p* > .05) (Figure 2c). Participants were also divided into two groups according to the mean TMS score of the cohort population. We found that there were significant difference between two groups on the scores of MMSE, total VFT, SDMT, Stroop reading/naming/interference, TMT, and HVAL‐R (*p* < .05) (Figure 2d).

Then, we used the variables that were statistically significant as predictors to conduct multiple linear regression models on six neuropsychological batteries (Stroop test, MMSE, TMT, VFT, SDMT, TMT, and HVLT‐R). Sex and age were forced to enter all models to adjust confounders. Our study revealed that age, TFC, education attainment, TMS, and AAO accounted for 54% of the variance in Stroop test assessment (*F* = 52.20, *p* < .001). In the model of MMSE, education attainment, TMS, TFC, and dysexecutive subdomain score of PBA‐s accounted for 60% of the variance in MMSE (*F* = 38.25, *p* < .001). The score of TMT could be accounted by sex, TMS, and irritability subdomain score of PBA‐s for 37% of the variance (*F* = 8.80, *p* < .001). The regression model of VFT test showed that TMS, education attainment, family history, and CAG accounted for 57% of the variance (*F* = 24.44, *p* < .001). SDMT could be accounted by TFC, AAO, education attainment, and TMS for 59% of the variance (*F* = 24.26, *p* < .001); and HVLT‐R could be accounted by CAG repeats, TFC, and education attainment for 41% of the variance (*F* = 7.80, *p* < .001) (Table 3).

**TABLE 3:** Regression model results showing the association between cognitive test performance (dependent variable) and variables of interest (independent variable) adjusted for sex and age.

Neuropsychological performance (domain/test)		Variables	Standardized coefficients beta	*p* Value	*F*	*R* Square
Executive function	Stroop test	Sex	−0.024	.635	*F* = 52.20 *p* < .001	.55 (Adjusted *R* ^2^: .54)
		Age	−0.234	<.001^a^		
		TFC	0.345	<.001^a^		
		Education attainment	0.351	<.001^a^		
		TMS	−0.269	<.001^a^		
		AAO	−0.119	.026^a^		
Global cognition	MMSE	Sex	0.034	.531	*F* = 38.25 *p* < .001	.61 (Adjusted *R* ^2^: .60)
		Age	−0.098	.085		
		Education attainment	0.363	<.001^a^		
		TMS	−0.142	.049^a^		
		TFC	0.346	<.001^a^		
		Dysexecutive	−0.227	<.001^a^		
Processing speed, executive function	TMT	Sex	−0.237	.019^a^	*F* = 8.80 *p* < .001	.41 (Adjusted *R* ^2^: .37)
		Age	−0.024	.953		
		AAO	0.355	.359		
		TMS	−0.488	<.001^a^		
		Irritability	−0.226	.034^a^		
Language	VFT	Sex	0.155	.080	*F* = 24.44 *p* < .001	.60 (Adjusted *R* ^2^: .57)
		Age	−0.139	.457		
		TMS	−0.544	<.001^a^		
		Education attainment	0.309	.001^a^		
		Family history	0.224	.007^a^		
		CAG	0.202	.023^a^		
Processing speed	SDMT	Sex	0.052	.679	*F* = 24.26 *p* < .001	.62 (Adjusted *R* ^2^: .59)
		Age	−0.823	.073		
		TFC	0.435	<.001^a^		
		AAO	−0.300	.001^a^		
		Education attainment	0.221	.012^a^		
		TMS	−0.267	.026^a^		
Episodic memory	Total Recall	Sex	0.052	.679	*F* = 7.80 *p* < .001	.47 (Adjusted *R* ^2^: .41)
		Age	−0.139	.457		
		CAG repeats	0.363	.012^a^		
		TFC	0.292	.040^a^		
		Education attainment	0.379	.004^a^		

Abbreviations: MMSE, mini–mental state examination; TFC, total functional capacity scale; TMS, total motor score in the unified Huntington's disease rating scale; SDMT, symbol digit modality test; TMT, trail making test.

^a^ *p* < .05, statistically significant.

## 4 DISCUSSION

The purpose of this study was to examine the cognitive status of the Chinese HD cohort and to explore the relevance of factors on sociodemographic and clinical characteristics, including motor and neuropsychiatric symptoms, to the cognitive assessment of different cognitive domains. We found that HD patients from China with a median disease duration of 4.5 years already had impairments in several cognitive domains at their first visit. Consistent with the previous studies (Abeyasinghe et al., 2021; Papoutsi et al., 2014), HD patients had deficits in their ability to multitask, their speed of processing, language, and executive function. Then, we explore the factors on sociodemographic and clinical features that could affect the function of diverse cognitive domains. Among these neuropsychological tests, our results revealed that the severity of motor symptoms, functional capacity, age, and AAO were correlated to all neuropsychological scores, whereas education attainment and diagnostic delay (duration from onset to diagnosis) were correlated to most neuropsychological scores except TMT. The severity of motor symptoms, functional capacity, and education attainment showed the predicting effect on diverse cognitive domains.

Cognitive decline, a marked and progressive symptom of HD phenotype, together with the motor sign, could be detectable even before the clinical diagnosis of HD (Papoutsi et al., 2014). Previous studies reported that in patients who are within 10 years from clinical diagnosis or “close to onset,” not only is there marked neurodegeneration but there are also subtle cognitive and motor deficits (Stout et al., 2012; Tabriz et al., 2013). In the study, we used six neuropsychological tests to test different cognitive domains, which were psychomotor speed (Dumas et al., 2013; Harrington et al., 2012) (tested by SDMT, TMT‐B, Stroop test), executive function (Dumas et al., 2013; Stout et al., 2011) (tested by Stroop Test, VFT, TMT‐B), language (tested by VFT), attention (Paulsen, 2011) (tested by MMSE, SDMT), and episodic memory (HVLT‐R). Our finding on HD patients from China was consistent with the previous findings in the Caucasian population (Tuijl et al., 2012) and the east Chinese population (Li et al., 2022). The score of MMSE of our HD patients showed mild cognitive impairment of patients, indicating deficits in registration (repeating named prompts), attention and calculation, recall, language, and ability to follow simple commands and orientation (Tuijl et al., 2012). Patients occurred semantic memory organization disorders, compared with the score of older healthy controls aged 60–69 (median [IQR]: 15 [19−22]) (Brody et al., 2019).

The first part of TMT is used primarily to examine cognitive processing and the second part in which the participant alternates between numbers and Chinese characters is used to examine executive functioning speed (Tombaugh, 2004). A previous study reported that an average score for TMT‐A is 29 s and a deficient score is greater than 78 s, and an average score is 75 s for TMT‐B and a deficient score is greater than 273 s. Thus, patients from our center obtained an evident lower score in executive functioning speed and cognitive processing on their first visit. Complementing and affirming the previous research (Papoutsi et al., 2014), diverse cognitive domains showed deficits across the whole stage of HD patients, even in the early stages of the disease. Therefore, early recognition and the longitudinal following up of cognition function in Chinese HD patients are important and essentially needed. Besides, the populations before clinical motor diagnosis should also be paid attention to. The latest HD integrated staging system would be recommended and help to facilitate the design of clinical trials targeting populations before clinical motor diagnosis (Tabriz et al., 2022).

In Pearson's correlation analyses, we identified factors that were correlated with the score of neuropsychological tests. We found that disease stage and the severity of motor symptoms were correlated with all cognitive test, and scores of MMSE and Stroop interference tend to be more related to patients’ behavior. Then, we used multiple linear regression to examine the relationship between clinical characteristics including motor and neuropsychiatric symptoms and cognitive function in patients with HD to determine the influencing factors related to different domains of cognitive function. We found that more education attainment, better TFC, and less severity of motor symptoms were associated with better global cognition and language function; younger age, the better TFC, more education attainment, and less severity of motor symptoms were associated with better executive function; male, less severity of motor symptoms, and irritability symptom were associated with better performance of TMT test; better TFC, later AAO, more education attainment, and less severity of motor symptom were associated with SDMT score; more CAG repeats, more education attainment, and better TFC were associated with better episodic memory they got. From the correlation analyses and six models of linear regression, we found the score of TMS showed a significant effect on cognitive domains of executive function, processing speed and language in all tests, which might suggest that motor symptoms are strongly associated with the cognitive function. However, the performance on cognitive tasks may be confounded with motor symptoms. In HD, impaired eye movements and speech production could negatively affect task performance, and these impairments may be indicative of motor function issues rather than cognitive deficits. Whereby the deficits in cognitive function might be related to severe motor symptoms, like the participants may not understand what they are instructed to do. In turn, cognitive dysfunction worsens motor performance, particularly on the more challenging items of the UHDRS–TMS. There are complex interactions within the motor, cortical, limbic, and oculomotor cortico‐basal ganglia‐thalamocortical networks arising in HD patients. There is likely a reciprocal interplay between motor function and cognitive abilities, during the disease progression. We may think that improving motor symptoms may be positive for cognitive function. Refining cognitive assessment measures in populations with movement disorders will not only provide deeper insights into patients’ capabilities but will also help elucidate the specific impact of striatal degeneration on prefrontal executive skills.

Education attainment was correlated to all tests and showed a significant effect in five models of linear regression, except for the TMT model. The previous study showed that the number of years of formal education completed by individuals is positively correlated with their cognitive function (Lövdén et al., 2020). We may think about whether prolonging education might affect the cognitive ability on gene‐mutation carriers if under good socioeconomic circumstances. Although the evidence of abnormal brain development in children and adolescents with the genetic expansion who manifested estimated after 35 years old (van der Plas et al., 2019), whether more education attainment could be the compensation to affect the cognition in the later manifested phase. Whether improving the conditions that shape development during the first decades of life carries great potential for improving cognitive ability in HD patients need to study further.

Behavioral dysfunctions, such as disorientation, apathy, affect, and irritability, are also needed to be early detected and treated to improve cognitive function. Our findings confirmed that the results from the previous research show correlations between objective cognition tested by MMSE and apathy (Baudic et al., 2006), apathy correlated with language function, memory, attention, and executive function tests. Irritability was also correlated with executive function tests. Additionally, depression could also be potential to effect the cognition. Therefore, it is important for the detection of neuropsychiatric symptoms. Furthermore, early evaluation and identification of cognitive decline are of keen interest in HD, where treatment with neuroprotective agents might delay the progression of cognitive decline. In our study, the adjusted *R* ^2^ values for models of Stroop, MMSE, TMT, VFT, SDMT, and HVLT‐R were large (.54, .60, .37, .57, .59, and .41, respectively) (Fritz et al., 2018).

Although our study provided valuable results, it did have some drawbacks. First, we could not determine causal relationships or moderating effects between variables, because the study was conducted using a cross‐sectional design. Therefore, additional longitudinal studies are needed to investigate the current findings. Second, when investigating the association between clinical features and cognitive function of Chinese HD patients, this study did not take the effect of treatment and disease duration into account. Third, the sample size of the study is relatively small, especially the sample of verbal learning test and also the control group is absent, so further expanding the sample size and recruitment of control group in the future to verify the results of the study is needed.

In conclusion, this study confirms that patients in China with HD had cognitive impairments in several cognitive domains in the early stage of the disease and that motor symptoms, education attainment, functional capacity, and behavioral dysfunction are independent risk factors in diverse cognitive domains. As a result, for a better prognosis, patients with HD require early assessment and recognition of cognitive impairments. The complex reciprocal interplay between motor function and cognitive abilities still remains to be studied further. Furthermore, our findings are useful in identifying risk factors on the cognitive function of patients with HD. Longitudinal monitoring of cognitive function in HD patients is needed and important.

## AUTHOR CONTRIBUTIONS

Conception and design; data analysis and interpretation; collect the data; drafting of the manuscript; revision for important intellectual content: Yang‐Fan Cheng. Conception and design; Collect the data: Kun‐Cheng Liu, Tian‐Mi Yang, and Yi Xiao. Conception and design; approving the final version of the manuscript that is to be published; accountability for all aspects of the work and the ability to identify the contributions of each coauthor and ensure the integrity of their contributions: Jean‐Marc Burgunder and Hui‐Fang Shang. Data analysis and interpretation; collect the data: Qi‐Rui Jiang, Jing‐Xuan Huang, and Sirui Zhang. Collect the data; drafting of the manuscript: Xiao‐Jing Gu.

## ETHICS APPROVAL AND CONSENT TO PARTICIPATE

Ethics approval for the study was approved by the institutional ethics committee of Sichuan University (approval number 2015‐236). Written informed consent was obtained from each participant or their primary caregiver.

## RELEVANT FINANCIAL RELATIONSHIPS

The authors report no financial or other relationship relevant to the participant of this article.

## CONFLICT OF INTEREST STATEMENT

The authors declare that they have no conflicts of interest.

### PEER REVIEW

The peer review history for this article is available at https://publons.com/publon/10.1002/brb3.3258.

## Supporting information

Table S1 Basic characteristics of the study population.
Table S2 The score of assessments on motor, neuropsychiatric symptoms, and cognitive function.

## Data Availability

Data and materials are available from the corresponding author on reasonable request.
