# Supplementary material for: Factors influencing cognitive function in patients with Huntington's disease from China: A cross‐sectional clinical study
Source: Brain Behav. 2023 Oct 17;13(11):e3258. doi: 10.1002/brb3.3258 (PMC10636378; doi:10.1002/brb3.3258)
Supplement: Supplementary file 1 — Table S1 Basic characteristics of the study population. Table S2 The score of assessments on motor, neuropsychiatric symptoms, and cognitive function. [file BRB3-13-e3258-s001.docx]

Supplementary table 1: Basic characteristics of the study population.

| **Variables** | **X ± S/N (%) / M (P25-P75)** | **Range** |
| --- | --- | --- |
| Sex (man%) | 84 (40.98%) |  |
| Age (years old) | 52.92 ± 11.54 | 21-83 |
| CAG repeats number | 44 (42-48) | 38-67 |
| Age of motor symptom onset (years old) | 41.81 ± 9.99 | 17-69 |
| Disease duration (years) | 4.5 (2.34-6.92). | 0-20 |
| Diagnostic delay (years) | 3.10 (1.84-5.74) | 0-20 |
| Positive family history | 162 (79%) | / |
| Education attainment (years) | 9 (6-12) | 0-19 |

M: median; S: standard deviation; X: mean.

Supplementary table 2: The score of assessments on motor, neuropsychiatric symptoms and cognitive function

|  | **Measures** | **Mean (SD)/ Median(P25-P75)** | **Range** |
| --- | --- | --- | --- |
| Motor and function assessment | TMS | 38 (27-48) | 9-83 |
|  | TFC | 9 (6-11) | 0-13 |
| Neuropsychiatric symptoms | HAMD | 10.19 (6.68) | 0-26 |
|  | BDI | 6.51 (5.90) | 0-29 |
|  | Affect in PBA-s | 4 (0-8) | 0-25 |
|  | Apathy in PBA-s | 1 (0-6) | 0-28 |
|  | Irritability in PBA-s | 2 (0-6) | 0-32 |
|  | Dysexecutive in PBA-s | 0 (0-0) | 0-16 |
|  | Psychosis in PBA-s | 0 (0-0) | 0-22 |
| Neuropsychological test | MMSE | 22.41 (5.32) | 7-30 |
|  | Verbal fluency Test | 9.97 (5.88) | 0-29 |
|  | SDMT | 18.52 (11.01) | 0-48 |
|  | Stroop Test | 95.18 (39.09) | 5-217 |
|  | Trail Making Test/ seconds | 273.1 (108.8) | 75-508 |
|  | TMT-A | 93 (67-144) | 27-268 |
|  | TMT-B | 170 (108-240) | 25-240 |
|  | Animal fluency | 9 (7-13) | 3-40 |
|  | HVLT-R | 17.85 (8.37) | 5-38 |
|  | Total Recall | 14.83 (5.75) | 5-29 |
|  | Delayed Free Recall | 5.17 (3.41) | 0-12 |

Abbreviations: BDI: Beck Depression Inventory; HAMD: Hamilton Depression Scale; PBA-s: short version of Problem-Behavior Assessment; TMS: total motor score in the Unified Huntington’s Disease Rating Scale; TFC: total functional capacity scale; MMSE: Mini-Mental State Examination; SDMT: Symbol Digit Modality test; HVLT-R: Hopkins Verbal Learning Test–Revised; TMT: Trail Making Task.
